# Validity of diagnostic codes used to ascertain maternal injuries during pregnancy

**DOI:** 10.1093/aje/kwaf145

**Published:** 2025-07-08

**Authors:** Asma M Ahmed, Riyan Deria, Rosalba Barojas Chavarria, Allie Sakowicz, David Stamilio, Elizabeth T Jensen

**Affiliations:** Department of Epidemiology and Prevention, Wake Forest University School of Medicine, Winston-Salem, NC, United States; Department of Obstetrics and Gynecology, Wake Forest University School of Medicine, Winston-Salem, NC, United States; Department of Pediatrics, Wake Forest University School of Medicine, Winston-Salem, NC, United States; Doctor of Medicine (MD) Program, Wake Forest University School of Medicine, Winston-Salem, NC, United States; Department of Epidemiology and Community Health, University of North Carolina (UNC) Charlotte, Charlotte, NC, United States; Department of Obstetrics and Gynecology, Wake Forest University School of Medicine, Winston-Salem, NC, United States; Department of Epidemiology and Prevention, Wake Forest University School of Medicine, Winston-Salem, NC, United States; Department of Obstetrics and Gynecology, Wake Forest University School of Medicine, Winston-Salem, NC, United States; Department of Epidemiology and Prevention, Wake Forest University School of Medicine, Winston-Salem, NC, United States

**Keywords:** maternal injuries, validation, ICD-10 codes, diagnostic codes, car accidents, falls, positive predictive value, PPV

## Abstract

Previous research has relied on International Classification of Diseases (ICD) codes to define maternal injuries. However, the validity of these codes remains unclear. We aimed to validate ICD-10 codes used to ascertain maternal injuries using medical chart reviews as the gold standard. A retrospective cohort study of all births occurring at Atrium Health Wake Forest Baptist Medical Center in 2022-2023. We randomly selected 100 subjects with ICD-10–indicated injury and 100 subjects without indication of injury. Two independent reviewers, blinded to the ICD-10–based classification, conducted the chart review. We examined the validity of relevant injury-related codes (V00-Y38; S00-T79; O9A.2-O9A.4) and calculated positive predictive values (PPVs) for different algorithms defined by varying the encounter type and the list of codes used. The algorithm that included all injury-related ICD-10 codes without encounter-type restrictions showed moderate PPV (71% [95% CI, 61%-79%]) and high negative predictive value (96% [90%-98%]). PPV was maximized when including codes V00-Y38 and restricting encounter type to inpatient or emergency department encounters (PPV 100% [93%-100%]). This study characterizes the accuracy of ICD-10–based algorithms for ascertaining maternal injuries during pregnancy. These findings can help improve inference by providing bias parameters for future research.

## Introduction

Injuries during pregnancy are relatively common events, affecting between 6% and 8% of pregnancies.^1^^-^^3^ Most maternal injuries are unintentional, and the 2 most common mechanisms are motor vehicle accidents and falls.^4^ Other mechanisms include exposure to mechanical forces, assaults, and intentional self-harm.^4^ Maternal injuries are the leading cause of nonobstetric-related deaths during pregnancy and the postpartum period.^1^^-^^4^ They are also associated with increased risks of multiple maternal and child adverse outcomes, including preterm birth, placental abruption, perinatal mortality and morbidity, and neurodevelopmental disorders in offspring.^1^^,^^4^^-^^21^

International Classification of Diseases, Tenth Revision, (ICD-10) codes have been utilized as the medical coding system in the United States since 2015.^22^ These codes are increasingly used in perinatal research because of their widespread use and accessibility.^23^^,^^24^ However, ICD-10 codes may not accurately reflect true medical conditions, as their primary purpose is for healthcare billing.^23^^,^^24^ Potential inaccuracies may arise due to diagnostic codes from previous encounters, rule-out diagnoses, or coding errors.^25^ Additionally, variations in coding practices across different healthcare systems may further contribute to inconsistencies in patient classifications. Therefore, the use of validated algorithms is crucial to reduce misclassification.

In the context of maternal injuries, large administrative data sources provide an important resource for studying the burden and impact of such injuries during pregnancy, as they provide access to large sample sizes at relatively low cost. Most previous research on maternal injuries has relied on clinical administrative databases and used ICD codes to identify injury-related events.^13^^,^^20^^,^^26^^-^^28^ Although studies have estimated the accuracy of ICD codes for specific injury types in the general population (eg, spinal cord injuries, traumatic brain injuries, and physical abuse),^29^^-^^34^ to our knowledge, no previous study has validated ICD-10 codes commonly used to ascertain injuries during pregnancy, either for injury as a broad category or for specific injury mechanisms. This retrospective cohort study aims to validate diagnostic codes used to ascertain maternal injuries, both for any injury and for the 2 most common mechanisms.

## Methods

### Setting

This population-based retrospective cohort study included women who gave birth (live or stillbirth, >20 weeks of gestation) at the Atrium Health Wake Forest Baptist Birth Center between January 1, 2022, and December 31, 2023, who had at least 1 prenatal care visit within the Atrium Health Wake Forest Baptist health system. Using ICD-10–based classification (algorithm 1 defined below), we classified women into injury cases and noninjury subjects, based on the presence of any of the injury-specific ICD-10 codes listed on any encounter (eg, inpatient, ED, outpatient, urgent care, and obstetric triage visits) between the start of pregnancy and delivery date. The start of pregnancy (ie, the date of the last menstrual period) was calculated based on the delivery date minus the gestational age at the time of delivery in days. Using the random number function in Excel, we then randomly selected 100 injury cases from those classified as injured and 100 noninjury subjects from those classified as noninjured based on ICD-10 classification for manual chart review. This study received ethics approval from Wake Forest University School of Medicine Institutional Review Board (IRB00103951) prior to its initiation.

### List of ICD-10 codes and algorithm definitions

We considered the following ICD-10 codes relevant to maternal injuries^35^: external causes of morbidity (V00-Y38); injury, poisoning, and certain other consequences of external causes (S00-T79); and traumatic injuries and abuse classifiable elsewhere but complicating pregnancy, childbirth, and the puerperium (O9A.2-O9A.4). We did not consider injuries related to medical or surgical errors or interventions. For injury as a broad category, we tested four different algorithms defined based on ICD-10 code lists and encounter types (Table 1). The first and second algorithms included all ICD-10 codes; the first included all encounter types (outpatient, inpatient, and emergency department [ED] encounters), while the latter was restricted to inpatient or ED encounters. We restrict some algorithms to inpatient and ED encounters (including obstetric triage) because (1) outpatient data could be less reliable due to the potential for historical diagnoses being carried forward to an outpatient encounter and the increased possibility of misclassification related to less severe or uncertain diagnoses being addressed in a lower acuity healthcare setting, and (2) several studies on maternal injuries only had access to inpatient and emergency department data,^8^^,^^14^^,^^20^^,^^27^ emphasizing the need to assess misclassification in these settings. We also tested algorithms that only included ICD-10 codes related to external causes of morbidities (V00-Y38) (algorithms 3 and 4). We then focused on transport accidents and falls, and tested codes specific to these 2 common injury mechanisms (algorithms 5-8).

**Table 1:** Description of ICD-10–based algorithms to define maternal injuries.

Name	Encounter type	ICD-10 codes
Algorithm 1 (any injury)	Any	**V00-Y38 (external causes of morbidity),** including transport accidents (V00-V99); Slipping, tripping, stumbling, and falls (W00-W19); Exposure to inanimate mechanical forces (W20-W49); Exposure to animate mechanical forces (W50-W64); Accidental drowning and submersion (W65-W74); Exposure to electric current, radiation, and extreme ambient air temperature and pressure (W85-W99); Exposure to smoke, fire, and flames (X00-X08); Contact with heat and hot substances (X10-X19); Exposure to forces of nature (X30-X39); Overexertion and strenuous or repetitive movements (X50-X50); Accidental exposure to other specified factors (X52-X58); Intentional self-harm (X71-X83); Assault (X92-Y09); Event of undetermined intent (Y21-Y33); Legal intervention, operations of war, military operations, and terrorism (Y35-Y38). **S00-T79 (injury, poisoning, and certain other consequences of external causes),** including Injuries to the head (S00-S09); Injuries to the neck (S10-S19); Injuries to the thorax (S20-S29); Injuries to the abdomen, lower back, lumbar spine, pelvis, and external genitals (S30-S39); Injuries to the shoulder and upper arm (S40-S49); Injuries to the elbow and forearm (S50-S59); Injuries to the wrist, hand, and fingers (S60-S69); Injuries to the hip and thigh (S70-S79); Injuries to the knee and lower leg (S80-S89); Injuries to the ankle and foot (S90-S99); Injuries involving multiple body regions (T07); Injury of unspecified body region (T14-T14); Effects of foreign body entering through natural orifice (T15-T19); Burns and corrosions of external body surface, specified by site (T20-T25); Burns and corrosions confined to eye and internal organs (T26-T28); Burns and corrosions of multiple and unspecified body regions (T30-T32); Frostbite (T33-T34); Poisoning by, adverse effect of, and underdosing of drugs, medicaments, and biological substances (T36-T50); Toxic effects of substances chiefly nonmedicinal as to source (T51-T65); Other and unspecified effects of external causes (T66-T78); Certain early complications of trauma (T79-T79). **O9A.2-O9A.4 (traumatic injuries and abuse classifiable elsewhere but complicating pregnancy, childbirth, and the puerperium),** including Injury, poisoning, and certain other consequences of external causes complicating pregnancy, childbirth, and the puerperium (O9A.2); Physical abuse complicating pregnancy, childbirth, and the puerperium (O9A.3); Sexual abuse complicating pregnancy, childbirth, and the puerperium (O9A.4).
Algorithm 2 (any injury)	Inpatient or ED encounters	
Algorithm 3 (any injury)	Any	**V00-Y38 (external causes of morbidity)**
Algorithm 4 (any injury)	Inpatient or ED encounters	
Algorithm 5 (transport accidents)	Any	**V00-V99 (transport accidents)**
Algorithm 6 (transport accidents)	Inpatient or ED encounters	
Algorithm 7 (falls)	Any	**W00-W19 (Slipping, tripping, stumbling, and falls)**
Algorithm 8 (falls)	Inpatient or ED encounters	

Abbreviations: ICD-10, International Classification of Diseases, Tenth Revision, (ICD-10) codes; ED, emergency department.

### The chart review process

Data obtained from manual abstraction of the electronic health record served as our gold standard for comparison. We used a system-wide unique patient identifier and date of birth to identify patients’ electronic health record. Health record abstractions were conducted by 2 independent reviewers (a medical student [R.D.] and a Master of Public Health student [R.B.]) who received training on the abstraction process by a postgraduate, Year 3 obstetrics and gynecology resident, with further input from other team members (perinatal epidemiologists and obstetricians). To minimize bias, the reviewers were blinded to the injury status of the patient during the reviews. In order to standardize the review process, results of the record review process were collected using a research electronic data capture (REDCap) form, adapted from Hansen *et al*.’s study.^36^ The REDCap form was used to document demographic characteristics about each subject, whether there was any injury-related encounter during pregnancy, and a description of the injury and encounter (eg, injury-associated symptoms and signs). For patients who experienced multiple injuries during their pregnancy, a separate REDCap form was completed for each injury. Each medical chart was reviewed by the 2 reviewers. Upon the completion of the abstractions, any inconsistency in the record review results was adjudicated by a third reviewer (A.A.). We limited our review to encounters during the period between the start of pregnancy (as defined above) and the delivery date. After comparing results of the record review and ICD-10–based classifications, electronic health records for all false-positive and false-negative injury cases were reviewed again by 2 additional reviewers (A.A. and A.S.) to determine the reason for discordant classification.

### Statistical analysis

We first summarized basic sociodemographic characteristics for those classified as injury cases or noninjury subjects. For algorithm 1, we calculated positive predictive values (PPVs—percentage of women classified as injury cases using the ICD-10–based algorithm who were confirmed as injured with record review as the gold standard) and negative predictive values (NPVs—percentage of women classified as not having an injury using the ICD-10–based algorithm who were accurately identified as uninjured using the gold standard) with 95% CIs. For the other algorithms, we only estimated PPV since the noninjury subjects were selected based on algorithm 1. For all false-positive and false-negative cases, we explored reasons why these women were misclassified based on ICD-10 algorithms. Statistical analyses were conducted with R version 4.2.3.

In additional analyses, we explored the validity of the ICD-10 codes included in the Center for Disease Control (CDC) external causes of injury matrix (V00–V99, W00–X58, ×71–X83, ×92–Y09, Y21–Y33, Y35–Y38, T14.91, T15–T19, T36–T50, T51–T65, T71, T73, T74, T75.0–T75.4, and T76) listed on any encounter.^35^^,^^37^^,^^38^

## Results

The study cohort comprised a total of 6297 births occurring at Atrium Health Wake Forest Baptist Medical Center during the study time period. From this cohort, we randomly chose 100 women from each group (those classified as injured and noninjured based on algorithm 1 defined above). Table 2 shows baseline characteristics for women included in each group. Compared to women classified as noninjury subjects, those classified as injury cases were more likely to be young (<20 years, 7% vs 4%), English-speaking (92% vs 82%), and non-Hispanic White (54% vs 47%).

**Table 2:** Demographic characteristics of women classified as injury cases or noninjured according to ICD-10–based classification.

Characteristics	Overall (*n* = 6297)	Noninjured (*n* = 100)	Injury cases (*n* = 100)
Maternal age			
<20 y	350 (6%)	4%	7%
20-24 y	1260 (20%)	23%	20%
25-29 y	1792 (29%)	25%	28%
30-34 y	1747 (28%)	25%	25%
35-39 y	913 (15%)	16%	17%
40+ y	235 (4%)	7%	3%
Race and ethnicity			
Hispanic or Latino	1762 (28%)	23%	22%
Non-Hispanic Black	1201 (19%)	25%	17%
Non-Hispanic White	2956 (47%)	47%	54%
Other	378 (6%)	5%	7%
Insurance status			
Public insurance	3513 (56%)	54%	54%
Private insurance	2731 (43%)	45%	44%
Self-pay	53 (1%)	1%	2%
Primary language spoken			
English	5260 (84%)	82%	92%
Spanish	935 (15%)	16%	8%
Other	102 (2%)	2%	0%

Notes: Overall cohort includes 2022-2023 births at Atrium Health Wake Forest Baptist Birth Center; Injury and noninjury subjects were selected based on algorithm 1 (ICD-10 codes: V00-Y38, S00-T79, and O9A.2-O9A.4; any encounter type).

Table 3 presents positive predictive values for all algorithms and negative predictive values for algorithm 1. Algorithm 1, which included all injury-related ICD-10 codes, showed moderate PPV (71% [95% CI, 61%-79%]) and high NPV (96% [95% CI, 90%-98%]). The PPV slightly improved after restricting encounter type to inpatient or ED encounters in algorithm 2 (PPV 85% [95% CI, 74%-91%]). Algorithms 3 and 4, which included ICD-10 codes related to external causes of morbidity—V00-Y38, with algorithm 4 additionally restricted to hospital or ED encounters—had very high PPV (97% [95% CI, 88%-99%] and 100% [95% CI, 97%-100%], respectively). Both algorithms focusing on transport-related accidents had very high PPV (96% [95% CI, 80%-99%] and 100% [95% CI, 83%-100%], respectively). Similarly for injury due to falls, algorithms 7 and 8 had near-perfect PPV.

**Table 3:** Validity of ICD-10–based algorithms for maternal injuries.

Algorithm	Brief description	Number of charts reviewed	PPV % (95% CI)	NPV % (95% CI) (100 charts reviewed)
Algorithm 1 (any injury)	ICD-10 codes: V00-Y38, S00-T79, and O9A.2-O9A.4; any encounter type	200	71% (61%-79%)	96% (90%-98%)
Algorithm 2 (any injury)	ICD-10 codes: V00-Y38, S00-T79, and O9A.2-O9A.4; inpatient or ED encounters	75	85% (76%-92%)	
Algorithm 3 (any injury)	ICD-10 codes: V00-Y38; any encounter type	59	97% (88%-99%)	
Algorithm 4 (any injury)	ICD-10 codes: V00-Y38; inpatient or ED encounters	51	100% (93%-100%)	
Algorithm 5 (transport accidents)	ICD-10 codes: V00-V99; any encounter type	24	96% (80%-99%)	
Algorithm 6 (transport accidents)	ICD-10 codes: V00-V99; inpatient or ED encounters	20	100% (84%-100%)	
Algorithm 7 (falls)	ICD-10 codes: W00-W19; any encounter type	29	100% (88%-100%)	
Algorithm 8 (falls)	ICD-10 codes: W00-W19; inpatient or ED encounters	27	100% (88%-100%)	

PPV = positive predictive value; NPV = negative predictive value.

Results using CDC injury codes were very similar to algorithm 3 (ICD-10 V00-Y38), with only 2 additional injury cases identified by the CDC algorithm and a PPV of 95% (87%–98%).

### Exploration of false positive and false negative cases

Algorithm 1 misclassified 29 women as injury cases despite the absence of any injury documentation upon reviewing medical charts (false-positive injury cases). A considerable proportion of these false positives (*n* = 11 [38%]) were related to codes or follow-up visits for injuries sustained before pregnancy (eg, previous fractures or surgeries). Another 7 cases were linked to muscle strains or back pain not associated with external injuries, and 5 were attributed to obstetric injuries during labor (eg, bladder injury during cesarean delivery). Three of the remaining false positives were related to spontaneous joint dislocations not caused by an injury (eg, shoulder dislocation), and another 3 were attributed to corneal abrasions. For women classified as noninjury subjects with algorithm 1, there were 4 false-negative cases. Three of these involved injury care received outside the health system, and the fourth was linked to a fall incident caused by a syncopal episode, which was coded as syncope.

With algorithm 2 (restricted to inpatient or ED encounters), there were 11 false-positive cases. Five of these were linked to obstetric injuries during labor (*n* = 5), and the remaining 7 were related to injuries sustained before pregnancy, muscle strains, or spontaneous joint dislocations.

With algorithm 3, there were only 2 false-positive cases; both were related to previous injuries sustained before pregnancy. Both false-positive cases were eliminated in algorithm 4, which was restricted to inpatient or ED encounters.

## Discussion

This retrospective cohort study examined the validity of diagnostic codes for ascertaining maternal injuries during pregnancy, both overall and for the 2 most common injury mechanisms. We compared several ICD-10–based algorithms to identify those with the highest PPV. Although all tested algorithms showed relatively high PPV, algorithms with a broader list of codes had the lowest PPV, while algorithms based only on ICD-10 codes for external causes of morbidities demonstrated the highest PPV (near 100%), minimizing false-positive rates.

Several previous studies have examined the accuracy of ICD codes for selected injury codes related to specific body regions in the general population,^31^^-^^33^ but to our knowledge, no previous study has focused on injuries during pregnancy. Hansen *et al*. validated ICD-10 codes O9A.2-O9A.4 (injuries complicating pregnancy, childbirth, and the puerperium) using EHR data from 1 Kentucky healthcare system between 2015 and 2017.^36^ This study did not focus solely on pregnancy but rather on any encounter that listed these codes (157 hospital or ED encounters). After restricting to injuries accompanied by potential obstetric complications (*n* = 113), PPV was 72.0% (95% CI, 65.0%-79.0%). These results are not directly comparable to ours, as these codes were rarely used in our data and were usually accompanied by other injury-specific codes. Out of the 100 women classified as injury cases using algorithm 1, only 5 injury encounters listed codes O9A.2-O9A.4; 4 of these had the obstetric-related codes alongside other injury-related codes.

PPV above 80%-90% is generally considered valid,^25^ suggesting that most algorithms we tested are valid for identifying injuries during pregnancy. Algorithm 1, which included a broader set of codes, may capture more true-positive injury cases, but it includes conditions not caused by external injuries, such as muscle strains and surgical complications, which decreases specificity. Restricting encounter types to hospital or ED encounters improved PPV (from 71% for algorithm 1 to 85% for algorithm 2). More restrictive algorithms that only included ICD-10 codes related to external causes of morbidity (V00-Y38) had near-perfect PPV, indicating very high specificity for these algorithms. In instances where the goal is to estimate incidence or prevalence, maximizing the NPV may be warranted. However, when maternal injury is the exposure or outcome of interest, maximizing PPV may be warranted, especially in the absence of any factor associated with both misclassification of injury and the exposure or outcome of interest. As an example, with injury as an outcome, more liberal definitions that maximize PPV may be justified, as relative risk estimations would be unbiased in the presence of nondifferential misclassification.^25^

### Strengths and limitations

This study was based on a cohort of all deliveries occurring at a birth center serving a diverse patient population. Our analysis was based on EHR data containing detailed information about all clinical encounters from pregnancy through delivery. We used standardized forms to capture all injury-related signs and symptoms, performed chart reviews independent of injury classification, and reviewed all false-positive and false-negative cases by additional reviewers, enhancing the rigor of our chart review process by minimizing chance of misclassification and adjudicator biases.

Our study has several limitations. First, the study included deliveries occurring at a single birthing center over a 2-year period, potentially limiting the generalizability of our findings, as coding practices and chart completeness may vary across institutions and over time. Therefore, larger studies based on diverse patient pools and healthcare settings are needed to validate these findings. Second, our study focused on predictive values, mainly on PPV, overlooking other metrics, such as sensitivity and specificity. PPV and NPV are influenced by the prevalence of injuries in the source population, which may limit their applicability to other settings. Larger studies that directly estimate the sensitivity and specificity of these codes are needed to help guide future research focusing on maternal injuries.

We used chart review as the gold standard, but this method has its limitations. In the absence of definitive diagnostic tests for injuries, we may have missed some injury cases, especially intentional injuries not disclosed when seeking care. For mechanism-specific algorithms, we may have misclassified some injury types, such as assault-related injuries misreported as falls. Furthermore, our chart review would miss any injury case that did not seek healthcare or those who sought care outside of our health system.

We only calculated NPV for algorithm 1; however, given the rarity of injuries during pregnancy, we expect that NPV to be relatively high across all algorithms. This study only considered clinical data to ascertain maternal injuries, but the use of alternative data sources, such as police reports for transport accidents or assault-related injuries, may help enhance injury detection.

### Conclusion

This study validates ICD-10 codes for ascertaining maternal injuries during pregnancy and compares the performance of several algorithms to identify those that can serve as reliable tools for future clinical and research applications. Our findings show that PPV can be optimized with more restrictive algorithms (selected ICD-10 codes, restricting to hospital or ED encounters). These findings offer important information for future research relying on administrative data and focusing on maternal injuries as a potential exposure or outcome. Because PPV and NPV depend on injury prevalence in the population, these results may be applicable for similar populations and may improve inference by providing bias parameters to inform quantitative bias analysis. Alternatively, studies could use predictive values to account for misclassification, provided they have additional information, such as true injury prevalence in the population or predictive values by exposure/disease status.^39^^-^^41^ Studies could also apply these predictive values in probabilistic bias analyses that incorporate uncertainty and varying assumptions.^39^ Further studies are needed to validate these codes in different healthcare settings and diverse populations, as are studies that directly estimate sensitivity and specificity to provide a more comprehensive assessment of the performance of these codes.

## Acknowledgments

Conference presentation: this work was presented as a poster presentation at the Society for Pediatric and Epidemiologic Research (SPER) annual meeting, Boston, MA, June 9-10, 2025.

## Data Availability

This study uses electronic health record (EHR) data from the Atrium Health Wake Forest Baptist Health system. Due to patient privacy and institutional policies, the data are not publicly available. Access may be granted with appropriate approvals.

## References

[1] Tinker SC, Reefhuis J, Dellinger AM, et al. Epidemiology of maternal injuries during pregnancy in a population-based study, 1997–2005. *J Womens Health*. 2010;19(12):2211-2218. 10.1089/jwh.2010.2160 21034174

[2] Harland KK, Saftlas AF, Yankowitz J, et al. Risk factors for maternal injuries in a population-based sample of pregnant women. *J Womens Health*. 2014;23(12):1033-1038. 10.1089/jwh.2013.4560 PMC4267121 25251144

[3] Osterman MJ, Hamilton BE, Martin JA, et al. Births: final data for 2022. *Natl Vital Stat Rep*. 2024;73(2):1-56. 10.15620/cdc:145588 38625869

[4] Mendez-Figueroa H, Dahlke JD, Vrees RA, et al. Trauma in pregnancy: an updated systematic review. *Am J Obstet Gynecol*. 2013;209(1):1-10. 10.1016/j.ajog.2013.01.021 23333541

[5] Vivian-Taylor J, Roberts CL, Chen JS, et al. Motor vehicle accidents during pregnancy: a population-based study. *BJOG.* 2012;119(4):499-503. 10.1111/j.1471-0528.2011.03226.x 22324920

[6] Schiff MA, Holt VL. Pregnancy outcomes following hospitalization for motor vehicle crashes in Washington state from 1989 to 2001. *Am J Epidemiol*. 2005;161(6):503-510. 10.1093/aje/kwi078 15746466

[7] Schiff MA, Holt VL, Daling JR. Maternal and infant outcomes after injury during pregnancy in Washington state from 1989 to 1997. *J Trauma Acute Care Surg*. 2002;53(5):939-945. 10.1097/00005373-200211000-00021 12435947

[8] Weiss HB, Sauber-Schatz EK, Cook LJ. The epidemiology of pregnancy-associated emergency department injury visits and their impact on birth outcomes. *Accid Anal Prev*. 2008;40(3):1088-1095. 10.1016/j.aap.2007.11.011 18460377

[9] El Kady D, Gilbert WM, Anderson J, et al. Trauma during pregnancy: an analysis of maternal and fetal outcomes in a large population. *Am J Obstet Gynecol*. 2004;190(6):1661-1668. 10.1016/j.ajog.2004.02.051 15284764

[10] Fabricant SP, Greiner KS, Caughey AB. Trauma in pregnancy and severe adverse perinatal outcomes. *J Matern Fetal Neonatal Med*. 2021;34(18):3070-3074. 10.1080/14767058.2019.1678129 31619114

[11] Schiff MA . Pregnancy outcomes following hospitalization for a fall in Washington state from 1987 to 2004. *Obstet Gynecol Surv*. 2009;64(4):215-216. 10.1097/01.ogx.0000345717.41262.0a 18947341

[12] Vladutiu CJ, Marshall SW, Poole C, et al. Adverse pregnancy outcomes following motor vehicle crashes. *Am J Prev Med*. 2013;45(5):629-636. 10.1016/j.amepre.2013.06.018 24139777 PMC3859429

[13] Chang Y-H, Chien Y-W, Chang C-H, et al. Maternal outcomes in association with motor vehicle crashes during pregnancy: a nationwide population-based retrospective study. *Inj Prev*. 2023;29(2):166-172. 10.1136/ip-2022-044810 36941051

[14] Pai C-W, Wiratama BS, Lin H-Y, et al. Association of traumatic injury with adverse pregnancy outcomes in Taiwan, 2004 to 2014. *JAMA Netw Open*. 2021;4(4):e217072-e217072. 10.1001/jamanetworkopen.2021.7072 33877308 PMC8058639

[15] Sperry JL, Casey BM, McIntire DD, et al. Long-term fetal outcomes in pregnant trauma patients. *Am J Surg*. 2006;192(6):715-721. 10.1016/j.amjsurg.2006.08.032 17161081

[16] Mehraban SS, Lagodka S, Kydd J, et al. Predictive risk factors of adverse perinatal outcomes following blunt abdominal trauma in pregnancy. *J Matern Fetal Neonatal Med*. 2022;35(25):8929-8935. 10.1080/14767058.2021.2007876 34852716

[17] Schuster M, Becker N, Young A, et al. Trauma in pregnancy: a review of the Pennsylvania Trauma Systems Foundation database. *Trauma.* 2018;20(1):30-37. 10.1177/1460408617703677

[18] Van Der Knoop B, Zonnenberg I, Otten V, et al. Trauma in pregnancy, obstetrical outcome in a tertiary centre in the Netherlands. *J Matern Fetal Neonatal Med*. 2018;31(3):339-346. 10.1080/14767058.2017.1285891 28395602

[19] Dalton SE, Sakowicz A, Charles AG, et al. Major trauma in pregnancy: prediction of maternal and perinatal adverse outcomes. *Am J Obstet Gynecol MFM*. 2023;5(9):101069. 10.1016/j.ajogmf.2023.101069 37399890

[20] Ahmed A, Rosella LC, Oskoui M, et al. In utero exposure to maternal injury and the associated risk of cerebral palsy. *JAMA Pediatr*. 2023;177(1):53-61. 10.1001/jamapediatrics.2022.4535 36441546 PMC9706397

[21] Chang Y-H, Chien Y-W, Chang C-H, et al. Neurodevelopmental disorders in children born to mothers involved in maternal motor vehicle crashes. *Pediatr Res*. 2024;97(5):1546-1553. 10.1038/s41390-024-03608-3 39349820

[22] CDC . International Classification of Diseases, Tenth Revision, Clinical Modification (ICD-10-CM). Atlanta, GA: CDC; 2023.

[23] Beam KS, Lee M, Hirst K, et al. Specificity of International Classification of Diseases codes for bronchopulmonary dysplasia: an investigation using electronic health record data and a large insurance database. *J Perinatol*. 2021;41(4):764-771. 10.1038/s41372-021-00965-3 33649436 PMC7917960

[24] Huybrechts KF, Bateman BT, Hernández-Díaz S. Use of real-world evidence from healthcare utilization data to evaluate drug safety during pregnancy. *Pharmacoepidemiol Drug Saf*. 2019;28(7):906-922. 10.1002/pds.4789 31074570 PMC6823105

[25] Straub L, Bateman BT, Hernandez-Diaz S, et al. Validity of claims-based algorithms to identify neurodevelopmental disorders in children. *Pharmacoepidemiol Drug Saf*. 2021;30(12):1635-1642. 10.1002/pds.5369 34623720 PMC8578450

[26] Chang Y-H, Chien Y-W, Chang C-H, et al. Associations of maternal motor vehicle crashes during pregnancy with offspring’s neonatal birth outcomes. *Int J Epidemiol*. 2023;52(6):1774-1782. 10.1093/ije/dyad125 37738433

[27] Cheng HT, Wang YC, Lo HC, et al. Trauma during pregnancy: a population-based analysis of maternal outcome. *World J Surg*. 2012;36(12):2767-2775. 10.1007/s00268-012-1750-6 22941234

[28] Liu S, Basso O, Kramer MS. Association between unintentional injury during pregnancy and excess risk of preterm birth and its neonatal sequelae. *Am J Epidemiol*. 2015;182(9):750-758. 10.1093/aje/kwv165 26409238

[29] Bågenholm A, Lundberg I, Straume B, et al. Injury coding in a national trauma registry: a one-year validation audit in a level 1 trauma centre. *BMC Emerg Med*. 2019;19(1):1-8. 10.1186/s12873-019-0276-8 31666018 PMC6820947

[30] Hlaing T, Hollister L, Aaland M. Trauma registry data validation: essential for quality trauma care. *J Trauma Acute Care Surg*. 2006;61(6):1400-1407. 10.1097/01.ta.0000195732.64475.87 17159683

[31] Noonan VK, Thorogood NP, Fingas M, et al. The validity of administrative data to classify patients with spinal column and cord injuries. *J Neurotrauma*. 2013;30(3):173-180. 10.1089/neu.2012.2441 23002989

[32] Warwick J, Slavova S, Bush J, et al. Validation of ICD-10-CM surveillance codes for traumatic brain injury inpatient hospitalizations. *Brain Inj*. 2020;34(13–14):1763-1770. 10.1080/02699052.2020.1849801 33280404 PMC7808251

[33] Welk B, Loh E, Shariff S, et al. An administrative data algorithm to identify traumatic spinal cord injured patients: a validation study. *Spinal Cord*. 2014;52(1):34-38. 10.1038/sc.2013.134 24216615

[34] Rasooly IR, Khan AN, Sierra MCA, et al. Validating use of ICD-10 diagnosis codes in identifying physical abuse among young children. *Acad Pediatr*. 2023;23(2):396-401. 10.1016/j.acap.2022.06.011 35777658 PMC10228836

[35] Hedegaard H et al. The International Classification of Diseases, 10th Revision, Clinical Modification (ICD–10–CM): External Cause-of-Injury Framework for Categorizing Mechanism and Intent of Injury, National Health Statistics Reports; n 136. 2019. Hyattsville, MD: National Center for Health Statistics. 32510317

[36] Hansen A, Quesinberry D, Akpunonu P, et al. Validation of ICD-10-CM codes for injuries complicating pregnancy, childbirth and the puerperium: a medical record review. *Inj Prev*. 2021;27(Suppl 1):i9-i12. 10.1136/injuryprev-2019-043512 33674327 PMC7948181

[37] Hedegaard H, Johnson RL, Garnett MF, Thomas KE. The 2020 International Classification of Diseases, 10th Revision, Clinical Modification Injury Diagnosis Framework for Categorizing Injuries by Body Region and Nature of Injury. National Health Statistics Reports; no 150. Hyattsville, MD: National Center for Health Statistics. 2020. Available from: https://www.cdc.gov/nchs/data/nhsr/nhsr150-508.pdf 33395385

[38] Hedegaard H, Johnson RL, Warner M, et al. Proposed framework for presenting injury data using the International Classification of Diseases, tenth revision, clinical modification (ICD-10-CM) diagnosis codes. National Health Statistics Reports; no 89. 2016. Hyattsville, MD: National Center for Health Statistics. 26828779

[39] Fox MP, MacLehose RF, Lash TL. Applying Quantitative Bias Analysis to Epidemiologic Data. Vol. 10. Cham: Springer; 2021.

[40] Brenner H, Gefeller O. Use of the positive predictive value to correct for disease misclassification in epidemiologic studies. *Am J Epidemiol*. 1993;138(11):1007-1015. 10.1093/oxfordjournals.aje.a116805 8256775

[41] Newcomer SR, Xu S, Kulldorff M, et al. A primer on quantitative bias analysis with positive predictive values in research using electronic health data. *J Am Med Inform Assoc*. 2019;26(12):1664-1674. 10.1093/jamia/ocz094 31365086 PMC6857512

